# Neuroendocrine-like cells -derived CXCL10 and CXCL11 induce the infiltration of tumor-associated macrophage leading to the poor prognosis of colorectal cancer

**DOI:** 10.18632/oncotarget.8423

**Published:** 2016-03-28

**Authors:** Yu-Jie Zeng, Wei Lai, Heng Wu, Lu Liu, He-Yang Xu, Jie Wang, Zhong-Hua Chu

**Affiliations:** ^1^ Guangdong Provincial Key Laboratory of Malignant Tumor Epigenetics and Gene Regulation, Department of Gastroenteropancreatic Surgery, Sun Yat-sen Memorial Hospital, Sun Yat-sen University, Guangzhou 510120, People's Republic of China

**Keywords:** colorectal cancer, neuroendocrine differentiation, tumor-associated macrophages, prognosis

## Abstract

Our previous study revealed that neuroendocrine differentiation in colorectal cancer is one of the important factors leading to worse prognosis. In this study, we apply immunohistochemical staining, Western-blot, RT-PCR and ELISA to investigate the underlying mechanism that how the neuroendocrine differentiation to affect the prognosis of colorectal cancer. The interaction of colorectal cancer cells, neuroendocrine-like cells and tumor-associated macrophages in colorectal cancer progress is also investigated. By analyzing 82 cases of colorectal cancer patients treated in our institution, we found that colorectal adenocarcinoma with neuroendocrine differentiation had increasing number of tumor-associated macrophages and worse prognosis. Further evaluation of cytology showed that neuroendocrine cells have the ability to recruit tumor-associated macrophages to infiltrate the tumor tissue, and the tumor-associated macrophages enhance the proliferation and invasion abilities of the colon cancer cells. Moreover, we confirmed that CXCL10 and CXCL11 are the key chemokines in neuroendocrine-like cells and they promote the chemotaxis activity of tumor-associated macrophages. The secretion of CXCL10 and CXCL11 by neuroendocrine-like cells can recruit tumor-associated macrophages to infiltrate in tumor tissues. The latter enhances the proliferation and invasion of colorectal cancer cell and lead to poor prognosis.

## INTRODUCTION

The phenomenon of neuroendocrine differentiation (NED) existed in neoplasia in a variety of non-neuroendocrine organs, including the gastrointestinal tract, prostate, lung and mammary gland [1–3]. Differentiated neuroendocrine cells are scattered as either a single cell or cell nests and are associated components of adenocarcinoma. In prostatic cancer research, it was shown that NED generally involves more aggressive clinical behavior and unfavorable prognoses [4–6]. The main characteristic of neuroendocrine cells in prostate cancer is that they can secrete product without relying on androgen to influence the tumor invasive behavior. They play an important role in the process of turning androgen sensitivity into androgen insensitivity in prostatic cancer. In colorectal cancer, other research [7] and ours [8] have shown that the emergence of neuroendocrine differentiation is accompanied by a worse prognosis of cancer. However, the possible underlying mechanism has not been reported.

Neuroendocrine differentiation is usually determined by the immunoreactivity of neuroendocrine markers, such as chromogranin A (CgA), and synaptophysin (Syn) [9]. Chromogranin A (CgA), a heat stable, hydrophilic acidic protein of about 460 amino acids, is a member of the granin family of secretory proteins that are ubiquitous to the nervous, endocrine and immune system [10, 11]. Synaptophysin (Syn), an integral membrane glycoprotein (polypeptide Mr 38000), is present in a variety of human neuroendocrine cells and neoplasms of both the neural and the epithelial type [12]. Based on the digestive tumor classification published by WHO in 2010 version 4, the joint detection of CgA and Syn is more reliable to confirm the neuroendocrine properties.

The study with mixed adenoneuroendocrine carcinoma (MANEC) showed that the neuroendocrine tumor cells and the adenocarcinoma cells were both derived from the same stem cell upon losing heterozygosity [13]. This observation was also supported by Modine et al [14]. Based on these reports, we were able to use colon cancer cell lines to construct neuroendocrine-like cells and investigate the possible mechanism between poor prognosis in colorectal carcinoma and neuroendocrine differentiation.

## RESULTS

### Neuroendocrine differentiation correlated with poor prognosis of colorectal adenocarcinoma

**Figure 1:**
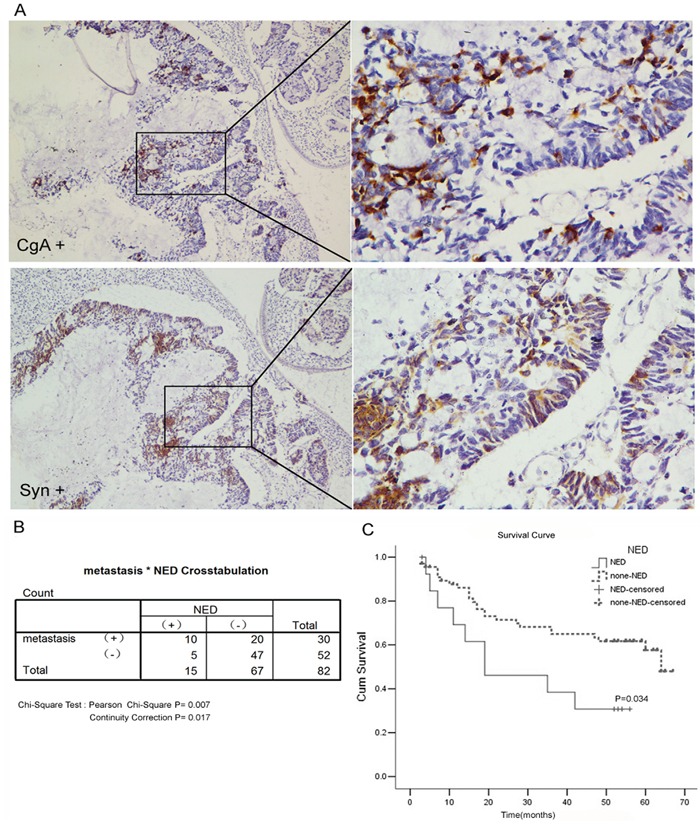
Neuroendocrine differentiation correlated with the poor prognosis of colorectal adenocarcinom **A.** The immunohistochemical staining of CgA or Syn. **B.** The rate of distant metastases in the NED group was significantly higher than that in the non-NED group (66.7% vs. 29.8%, P=0.007). **C.** Kaplan-Meier survival curve of patients between NED group and non-NED group.

A total of 82 cases with colorectal adenocarcinoma were followed. All these patients had received no pre-operation chemotherapy. They were given the same radical operation in 2008 and underwent the same adjuvant chemotherapy (the chemotherapy regimen is oxaliplatin combined with 5-Fu and leucovorin) after the surgery, all tumor samples were paraffin-embedded and sections were re-stained for CgA and Syn using immunohistochemisry (Figure 1A).

Among these tumor samples, fifteen cases showed positive CgA and/or Syn expression (Table 1) and these cases were referred as the NED group. The remaining 67 cases were the non-NED group. Ten cases (66.7%) in the NED group developed distant metastases during follow up: 8 cases were liver metastases, 1 case was lung metastasis and 1 case was bone metastasis. Twenty cases (29.8%) developed distant metastases in the non-NED group, which was significantly different from the NED group (Chi-square test *X^2^*=7.16, P=0.007). The 5-year survival rate was 26.7% (4/15) in the NED group and 52.2% (35/67) in the non-NED group. These rates were significantly different (Chi-square test *X^2^*= 4.506, P=0.034) (Figure 1B and 1C).

**Table 1:** The clinical information of the 15 colorectal cancer patients with CgA and/or Syn stained positive

NO.	Sex	Age	Tumor site	TNM stage	Tumor differentiation	Site of metastasis	Time of metastasis	Following up result
1	Male	51	Transverse colon	IIIB	Well	Liver	8 months after operation	Dead, 14 months survival
2	Female	69	Ascending colon	IIIB	Poor	Liver	31 months after operation	Dead, 42 months survival
3	Female	59	Sigmoid colon	IIA	Well	Liver	13 months after operation	Dead, 19 months survival
4	Male	47	Ascending colon	IIA	Moderately	None	NA	Live, follow-up 56 months
5	Male	56	Ascending colon	IIA	Moderately	None	NA	Live, follow-up 54 months
6	Female	66	Ascending colon	IIA	Moderately	None	NA	Live, follow-up 53 months
7	Female	69	Sigmoid colon	IIA	Well	None	NA	Live, follow-up 52 months
8	Female	56	Sigmoid colon	IIIB	Moderately	Liver	6months after operation	Dead, 11 months survival
9	Male	56	Rectum	IV	Well	Lung	Before the operation	Dead, 16 months survival
10	Male	57	Rectum	IIIB	Moderately	Liver	3months after operation	Dead, 7 months survival
11	Male	73	Rectum	IIIC	Well	Liver	17 months after operation	Dead, 19 months survival
12	Female	66	Sigmoid colon	IIIB	Moderately	Lost to follow-up	NA	Lost to follow-up >7 months survival
13	Male	72	descending colon	IIB	Well	vertebra	29 months after operation	Dead, 35 months survival
14	Female	41	Rectum	IV	Poor	Liver	Before the operation	Dead, 5 months survival
15	Male	46	Ascending colon	IV	Poor	Liver	Before the operation	Dead, 4 months survival

Upon comparing the TMN stage and tumor differentiation, which are the important indices in evaluating colorectal adenocarcinoma prognosis, we did not find a significant difference in the distribution between the groups (Table 2). And the multivariate Cox Regression indicated that neuroendocrine differentiation is a prognostic indicator independent of the tumor stage or tumor differentiation (HR=2.576, 95%CI 1.162-5.711, P=0.02). Therefore, our data indicated that the colorectal adenocarcinoma with neuroendocrine differentiation has higher invasion ability and a worse prognosis.

**Table 2:** Baseline data with neuroendocrine differentiation

Factor	Case				
	Total	NED	Non-NED	X^2^	P
All patients	82	15	67		
Sex				0.006	0.939
Male	43	8	35		
Female	39	7	32		
Age				0.006	1.000
<50	17	3	14		
>50	65	12	53		
Tumor Site				0.221	0.765
Colon	56	11	45		
Rectum	26	4	22		
Tumor Differentiation				0.111	0.946
High	31	6	25		
Moderate	32	6	26		
Low	19	3	16		
TNM Stage				2.999	0.392
I	6	0	6		
II	28	6	22		
III	36	6	30		
IV	12	3	9		
Lymphatic metastasis				0.106	0.899
Positive	48	9	39		
Negative	34	6	28		
Distant Metastasis				7.16	0.007
Yes	30	10	20		
No	52	5	47		

### The association of neuroendocrine and tumor-associated macrophages in colorectal adenocarcinoma tissue

**Figure 2:**
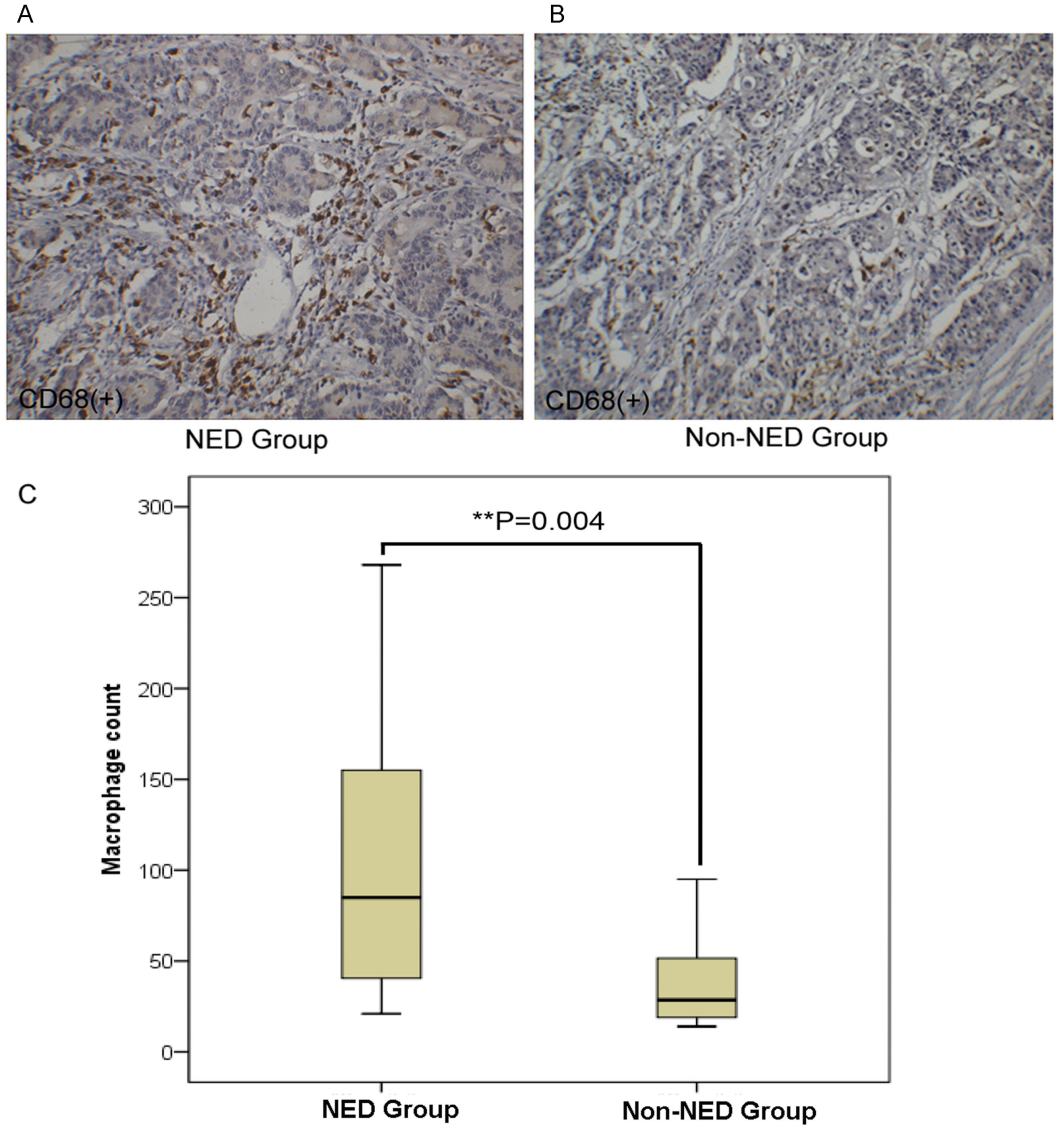
Correlation of neuroendocrine and tumor-associated macrophages in colorectal adenocarcinoma tissue **A-B.** The immunohistochemical staining of tumor-associated macrophages (stained by CD68). **C.** Compared with the non-NED group, the number of tumor-associated macrophage increased in the NED group. Bars correspond to the mean ± SD.

**Figure 3:**
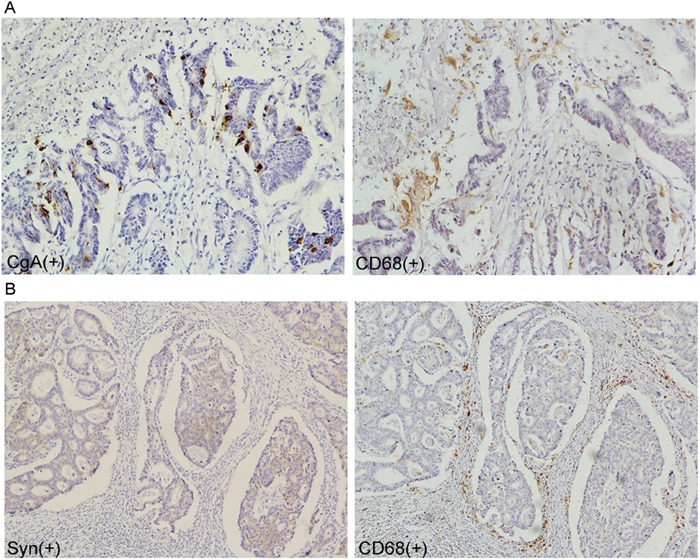
Correlation of neuroendocrine and tumor-associated macrophages in colorectal adenocarcinoma tissue **A-B.** tumor-associated macrophages crowded around the neuroendocrine cell showed by immunohistochemical staining of the serial section.

Compared with the non-NED group, the tumor-associated macrophages (TAMs, stained by CD68) count in the NED group increased (Figure 2). Further, the immunohistochemistry stain from the serial section of the colorectal adenocarcinoma samples showed that the neuroendocrine markers were expressed where neuroendocrine cells emerged and tumor-associated macrophages aggregated (Figure 3). Macrophages were counted by selecting 5 fields at high magnification (400X) and the average number of cells in each field was calculated.

This finding indicated that neuroendocrine cells may have the ability to recruit tumor-associated macrophages into tumor tissue.

### Chemotaxis of neuroendocrine-like cells for tumor-associated macrophages (TAMs)

**Figure 4:**
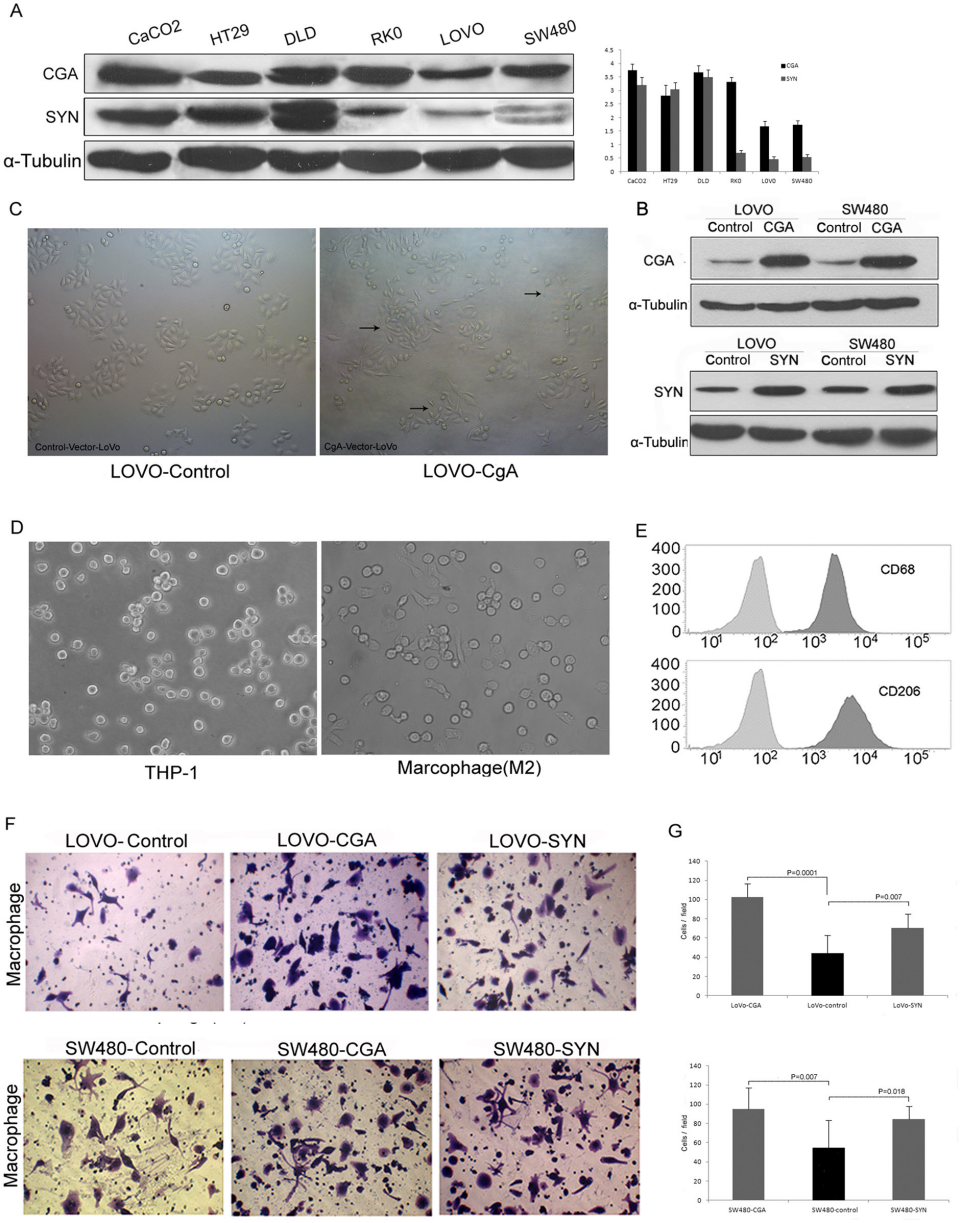
Chemotaxis of neuroendocrine-like cells for tumor-associated macrophages **A.** The expression of CgA and Syn in the 6 colon cancer cell lines. Bars correspond to the mean ± SD. **B.** Western blot was used to verify CgA or Syn expression in the stable colon cell lines. **C.** The morphological change of the neuroendocrine-like cell (LOVO-HA-CgA), which was similar to the tentacle-like protuberance of nerve cell. **D.** Normal conditions of THP-1 (left), and treated with PMA 320 nM for 6 h and then added IL-4 20 ng/ml for 18 h (right). **E.** The THP-1 showed significant induction for CD68 (for macrophages differentiation) and CD206 (for TAMs/M2 macrophages). **F.** Transwell assay was used to compare the TAM migration ability between LOVO/SW480-CgA or LOVO/SW480-Syn cells with LOVO/SW480-control cells. **G.** The numbers of cells passed through the Matrigel matrix. Bars correspond to the mean ± SD.

According to the cell homology theory stated above, we evaluated 6 colon cancer cell lines (Caco2, DLD1, HT-29, LOVO, RKO, and SW480) by Western blot to determine the expression of CgA and Syn (Figure 4A). According to this Western blot results, we selected LOVO and SW480 and over-expressed the CgA and Syn genes to construct the neuroendocrine-like cells because of their relatively naturally low expression of CgA and Syn. Western blot was used to verify CgA expression in CgA-Vector-LOVO/SW480 cells (LOVO-HA-CgA / SW480-HA-CgA) and Control-Vector-LOVO (LOVO-Control) cells, as occurred when determining Syn expression (Figure 4B). The LOVO-HA-CgA cells showed irregular dendrite-like processes that are typical of NE cells, but there was no signigicant morphological change in the LOVO-Control cells (Figure 4C).

M2 macrophages are a type of TAMs that are activated by the interleukin-4 (IL-4) produced by CD4^+^-T cells. Human THP-1 cells are often used for macrophage differentiation. THP-1 cells were suspended under normal conditions and then treated with phorbol myristate acetate (PMA) (320 nM/1×10^6^ cells) for 6 h. Finally, IL-4 (20 ng/ml) was added for 18 h (total of 24 h) [15]. The cells became larger and attached and exhibited pseudopodia. (Figure 4D). Moreover, the PMA-treated THP-1 cells expressed CD68 and CD206, which are two significant surface markers of TAMs (M2 macrophages) (Figure 4E).

We compared the TMA migration ability of the LOVO/SW480-HA-CgA and LOVO/SW480-HA-Syn cells with that of the LOVO/SW480-control cells using a transwell assay. When co-cultured with these neuroendocrine-like cells (located in the lower chamber) for 48 h, the migration ability of the TAMs was enhanced (Figure 4F and 4G)

These cytological results illustrated that the neuroendocrine-like cells have stronger chemotaxis signal for tumor-associated macrophages.

### Tumor-associated macrophages enhanced proliferation and invasion of colon cancer cells *in vitro*

**Figure 5:**
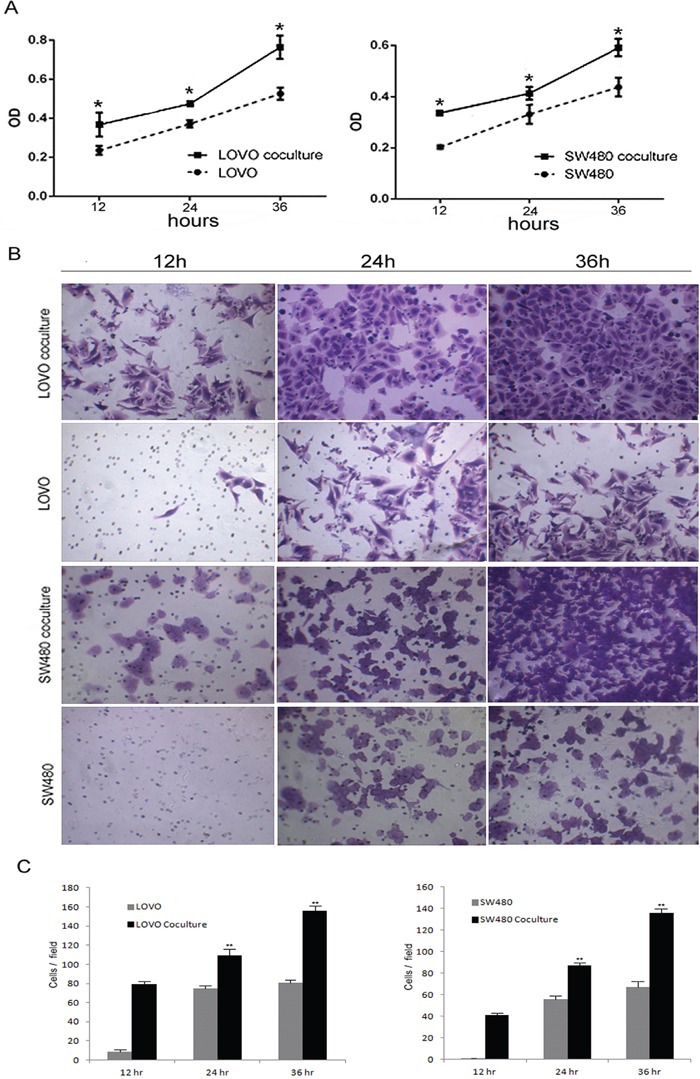
Tumor-associated macrophages enhanced proliferation and invasion of colon cancer cells **A.** Compare to the colon cells without co-culture with TAMs, the proliferation ability of the cell co-culture with TAMs was increased. Bars correspond to the mean ± SD. **B.** Transwell chamber assays for colon cells after cocultured with or without TAMs for 12, 24, 36 h, respectively. **C.** The numbers of cells passed through the Matrigel matrix. Bars correspond to the mean ± SD.

We co-cultured LOVO or SW480 cells with TAMs using a transwell assay. We found that the TAMs enhanced the proliferation and invasion ability of the colon cancer cell lines. (Figure 5).

Based on these findings, we speculated that neuroendocrine cells may recruit tumor-associated macrophages to infiltrate the colorectal cancer tissue, which accelerates colorectal cancer progression. This might be the cause for the worse prognoses in colorectal adenocarcinoma with neuroendocrine differentiation.

### Neuroendocrine-like cells promote the chemotaxis activity of TAM via CXCL10 and CXCL11

**Figure 6:**
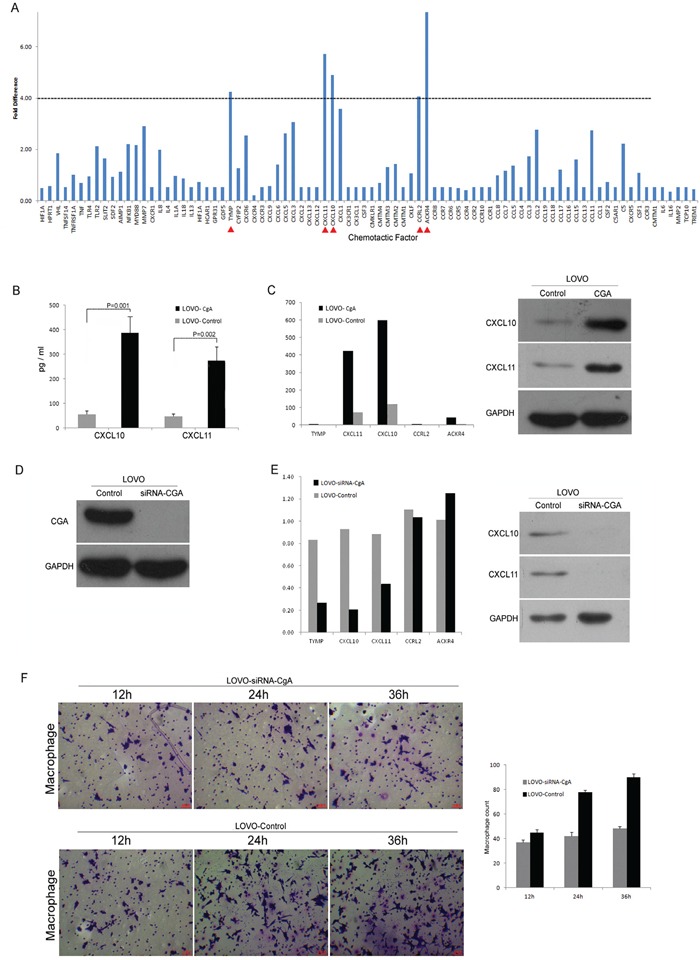
Neuroendocrine-like cells promote the chemotaxis activity of TAM via CXCL10 and CXCL11 **A.** The Neuroendocrine-like cells for chemokine family screening by RT-PCR. **B.** ELISA was used to confirm that the CXCL10 and CXCL11 increased obviously in the supernatant of neuroendocrine-like cell (LOVO-HA-CgA). Bars correspond to the mean ± SD. **C.** Western blot and RT-PCR was used to confirm that the CXCL10 and CXCL11 increased obviously in the neuroendocrine-like cell (LOVO-HA-CgA). **D.** Western blot was used to verify the knocking down of CgA in the LOVO cells by SiRNA. **E.** Western blot and RT-PCR was used to confirm that the CXCL10 and CXCL11 decreased obviously in the colon cell which was knocked down of CgA (LOVO-SiRNA-CgA). **F.** Transwell assay was used to compare the migration ability between LOVO-SiRNA-CgA with LOVO-control cells, and the numbers of cells passed through the Matrigel matrix. Bars correspond to the mean ± SD.

We evaluated the LOVO-HA-CgA cells for chemokine family using RT-PCR. The result showed that five markers (CXCL10, CXCL11, TYMP, ACKR4, and CCRL2) were increased more than 4-fold (Figure 6A). And then, We further evaluated the effect of LOVO-HA-CgA cells and LOVO-SiRNA-CgA cells (knocked down CgA in the LOVO cells By SiRNA) (Figure 6D) by RT-PCR, Western blot (Figure 6C and 6E) and ELISA (Figure 6B). We confirmed that CXCL10 and CXCL11 are key chemokines in the neuroendocrine-like cells that promote the chemotaxis activity of TAM. When we co-cultured the LOVO-siRNA-CgA cells, the migration ability of the TAMs was weakened (Figure 6F), compared to the LOVO-Control cells.

## DISCUSSION

To date, there is no uniform criterion for diagnosing and classifying neuroendocrine differentiation in colorectal adenocarcinoma. There were four different classification criteria in this research field as showed in our published Meta-analysis [8]. A total of 11 articles from 1992 to 2010 were included in our paper. However, the mixed adenoneuroendocrine carcinoma (MANEC), which was first named by *2010 WHO classification of tumors of the digestive system* [16], consisted of both adenocarcinoma and neuroendocrine carcinoma cells and the proportions of either one need to be more than 30 percent, was brought up in the year of 2010. Coupled with that, there are approximately 2 percent of neuroendocrine cells in normal colorectal epithelium cells or in the colorectal cancer tissue [17]. Therefore, we deduced such criteria from these published studies and proposed that neuroendocrine differentiation in colorectal adenocarcinoma could be defined by a proportion of positive neuroendocrine differentiation indicators ranging from 2% to 30%. We classified the 82 cases of neuroendocrine differentiation based on this premise.

The effect of neuroendocrine differentiation on the prognosis of colorectal cancer is controversial in the literature. Therefore, we performed a meta-analysis and summarized the 82 cases in our hospital to determine whether neuroendocrine differentiation is one of the important factors leading to worse prognosis of colorectal cancer. Our study indicated that the tumor with neuroendocrine differentiation had higher tumor invasion ability and a greater number of metastases. In contrast to these literatures, we used both neuroendocrine markers, CgA and Syn, to determine the presence of neuroendocrine differentiation. Moreover, we set a standard range for evaluation to exclude the influence of MANEC or neuroendocrine tumors. The 82 cases had radical operations that were performed by the same treatment group with consistent follow-up therapy. This reduced the influence of confounding factors and ensured reliable conclusions. We had excluded cases with pre-operation chemotherapy, because the report by Jinru Shia [18] showed that there was an increased frequency and density of cells with a neuroendocrine phenotype in rectal adenocarcinomas that were subjected to neoadjuvant therapy and that the extent of neuroendocrine cells appears proportional to the degree of treatment response.

As all we know, colorectal cancer patients with stage II (TNM stage) will be determined for adjuvant chemotherapy according to the risk factor, including poorly differentiated histology (exclusive of those cancers that are MSI-H), lymphatic or vascular invasion, and bowel obstruction, less than 12 lymph nodes examined, perineural invasion, localized perforation or close, indeterminate or positive margins, etc. And then, the neuroendocrine differentiation also could be considered for a risk factor in the prognosis assessment strategies of colorectal cancer through our studies.

The possible mechanism for the effect of neuroendocrine differentiation on colorectal cancer prognosis had not been reported. It is often reported that TAMs in the tumor microenvironment along with their secreted cytokines, such as IL-6, interact with prostate cancer cells and play an important role in neuroendocrine differentiation and the prognosis of prostate cancer [19, 20]. Therefore, we attempted to verify the possible association between neuroendocrine differentiation in colorectal cancer and the tumor microenvironment in this study. The tumor microenvironment comprises a variety of nonmalignant stromal cells that play pivotal roles in tumor progression and metastasis [21]. Among them, TAMs are the most notable migratory hematopoietic cell type [22, 23]. Evidence from clinical and epidemiological studies has shown a strong association between TAM density and poor prognosis in several types of cancer, including colorectal cancer. TAM-associated inflammation is known as the seventh hallmark of cancer [24, 25]. Our study showed that TAMs were gathered in the tissue of colorectal cancer with neuroendocrine differentiation and, in particular, surrounded the neuroendocrine cells. There are two types of TAMs. M1 macrophages are linked to antitumor activity, whereas M2 macrophages are associated with cancer progression and metastasis [26]. M2 macrophages express the surface markers CD68 and CD206 and contribute to tumor progression by releasing a variety of cytokines, including chemokines, inflammatory factors, and growth factors [24, 27]. Our study also proved that TAMs enhanced the proliferation and invasion ability of the colon cancer cell lines in the co-culture system. Therefore, we may speculate that neuroendocrine cells could recruit more tumor-associated macrophages to infiltrate the colorectal cancer tissue and thereby accelerate the progression of colorectal cancer.

However, there was no neuroendocrine cell available commercially. In the prostate cancer studies [4, 19, 28, 29], the authors took the method of transforming prostate cancer cells into neuroendocrine differentiation cell for research. In colorectal cancer, Modine et al [14] showed that the intestinal epithelial cells and neuroendocrine cells derived from the same stem cells. By analyzing the loss of heterozygosity between epithelial adenocarcinoma cells and neuroendocrine tumor cells in the mixed adenoneuroendocrine carcinoma, Furlan D et al [13] found that these two kinds of cells were homology. Based on these literatures, we assumed that colon cancer cells could be transformed into neuroendocrine cells by overexpressing the neuroendocrine differentiation markers CgA and Syn.

We used PMA to induce the transformation of THP-1 monocytes into M2 macrophages according to previously described methods [15]. We then chose the colon cell lines LOVO and SW480 to construct CgA- and Syn-expressing stable cell lines. These CgA- and Syn-expressing stable cell lines simulated neuroendocrine-like cells with their cell homology. They had a strong chemotaxis effect on the M2 tumor-associated macrophages. Because CgA is the most specific indicator for diagnosing neuroendocrine features, the abundance of LOVO-HA-CgA is higher than SW480-HA-CgA. We used LOVO-HA-CgA to screen the common chemokine family by RT-PCR and evaluate the expression levels of chemokine. The results showed five elevated chemotactic factors, including CXCL10, CXCL11, TYMP, CCRL2, and ACKR4. Furthermore, we used Western blot, ELISA and cytokine profiles to verify these chemotactic factors. We also used siRNA technology to verify the chemotactic relationship. The results showed that CXCl10 and CXCL11 were the key chemotactic factors in the interaction of TAMs and neuroendocrine differentiated cells.

It is well known that the chemokines CXCL10 and CXCL11 play an important role on the recruitment and activation of T cells, B cells, mononuclear macrophage, dendritic cells, NK cells and other immune inflammatory cells. Recently, studies have shown that CXCL10 over-expression is a distinct gene signature of acute-phase graft injury and tumor invasiveness in small-for-size liver grafts and that it can induce EPC mobilization, differentiation and neo-vessel formation, which further promotes tumor recurrence after liver transplantation [30, 31]. Studies have also found significantly higher CXCL10 protein levels in cancer tissue than in normal paired tissue. Elevated serum CXCL10 levels were significantly associated with poor survival in all stages or in stage I-III with curative patients and were an independent marker in predicting liver metastasis [32, 33]. Our finding showed that CXCL10 and CXCL11 play important roles in the interaction of neuroendocrine differentiation and TAMs and the poor prognosis of colorectal cancer. This result is the first to demonstrate a possible mechanism for the poor prognosis in colorectal cancer with neuroendocrine differentiation. Future study will focus on the exact signaling pathways related with neuroendocrine differentiation.

## MATERIALS AND METHODS

### Sample and patients

Colorectal cancer samples were obtained from 82 patients, who were admitted to the department of Gastroenteropancreatic Surgery of Sun Yat-sen Memorial Hospital, Sun Yat-sen University in 2008. Surgically resected specimens were collected immediately after tumor removal. The protocol was approved by the Ethics Committee of Sun Yat-Sen memorial hospital.

### Cell cultures and reagents

Six colon cell lines (i.e., Caco2, DLD1, HT-29, LOVO, RKO, SW480) and 293T cell line, purchased from the Shanghai Cell Bank of the Chinese Academy of Sciences, were cultured in Dulbecco's Modification of Eagle's Medium (DMEM; Gibco, Carlsbad, CA, USA) supplemented with 10% fetal bovine serum (FBS; Gibco, Carlsbad, CA, USA). The stably transfected cell lines were cultured in DMEM supplemented with 10% FBS and 1 μg/mL puromycin (Sigama-Aldrich, St.louis, MO, USA). All cells were maintained at 37°C in a humidified incubator with 5% CO_2_. G418, PMA, Lipofectamine2000 were purchased from Sigma (USA), RPMI-1640 was purchased from Gibco (USA), Trizol Reagent and Prime Script RT reagent were purchased from TAKARA (Japan), Matrigel was purchased from BD (USA), siRNA was purchased from GenePharma (China). Antibodies against CgA were purchased from Chemi-Con, antibodies against Syn were purchased from Cell signaling, antibodies against CXCL10 and CXCL11 were purchased from Abcam, Antibodies against CD68 and CD206 were purchased from Santa Cruz. THP-1 cells were obtained from Shanghai Cell Bank of the Chinese Academy of Sciences. M2-polarized THP-1 was generated by PMA (320 nM/10^6^ cells) for 6 h and then added IL-4 for another 18 h.

### Production of CgA and Syn stable colon cell lines

PCR fragments of HA-CgA and HA-Syn were inserted into BgiII and XhoI sites of the pMSCV-PIG vector. LOVO and SW480 cell lines were stably infected using pMSCV-PIG vector containing the human CgA or Syn gene. HEK293T packaging cells were transfected with the appropriate retroviral construct using Lipofectamine 2000 (Invitrogen). Culture supernatants were collected 36 to 60 hours after transfection and filtered. Target cells were infected with the filtered viral supernatants in the presence of 6 μg/mL Polybrene for 48 hours, after which the medium was changed. Following infection, cells were selected with 4 μg/mL puromycin for 2 weeks, and the resistant population was used for cellular assays.

### Immunohistochemistry (IHC)

The immunohistochemical study of CgA, Syn and CD68 was performed using a standard two-step technique. Paraffin sections were dried for 20min at 68°C, dewaxed in xylene, rehydrated through graded alcohol, and immersed in 3% hydrogen peroxide for 15 min to block endogenous peroxidase activity. An antigen retrieval process was accomplished using hyperbaric heating (HH) repair with 10 mM citrate buffer (pH6) for 3 mins. The slides were incubated with 5% normal goat serum at room temperature for 30 min to reduce nonspecific reaction. Subsequently, the slides were incubated overnight at 4°C with rabbit polyclonal antibody against CgA (1:100; Chemi-Con, German) or rabbit monoclonal antibody against Syn (1:100; Cell Signaling Technology, USA) or rabbit monoclonal antibody against CD68 (1:100; Santa Cruz, USA). After rinsing three times with 0.01 mol/L phosphate-buffered saline (PBS; pH = 7.4) for 10 mins, the detection of the primary antibody was achieved by addition of a secondary antibody (Envision; Dako, Glostrup, Denmark) for 1 hr at room temperature, and stained with DAB (3,3-diaminobenzidine) after washing in PBS again. Finally, the sections were counterstained with Mayer's hematoxylin, dehydrated, and mounted. PBS replaced primary antibody was used as a negative control.

### Flow cytometry

Cells were PBS washed and resuspended and then stained with murine antihuman CD68 or CD206 for 30min, then washed and incubated with PE-conjugated goat anti-mouse secondary antibody. After being washed, cells were analyzed by flow cytometry.

### Enzyme linked immunosorbent assay (ELISA)

ELISA was used to detect CXCL10 or CXCL11 in the culture supernatant of LOVO-HA-CgA and LOVO-HA-control cells by using Quantikine Kit (R&D Systems, Minneapolis, MN) according to the manufacturer's protocol. The product of the enzymatic reaction is yellowish and absorbs at 450 nm. The intensity of the yellowish refers to the amount of CXCL10 or CXCL11 in the culture supernatant.

### Western blot assay

Cells were harvested and lysed in cell lysis buffer (50 mM Tris-HCl [pH 7.4], 250 mM NaCl, 0.1% NP- 40, 5 mM EDTA, 2 ug/ml leupeptin, 2 ug/ml aprotinin, 4 mM Prefabloc SC, and protein inhibitor cocktail). Each 25 ug aliquot of denatured protein were separated by 10% SDS-PAGE, and then transferred onto a 0.22 um polyvinylidene difluoride membranes (Millipore). After completing protein transfer, the membrane was blocked in 5% (w/v) skimmed milk in TBST and incubated overnight at 4°C with the rabbit polyclonal antibody (IgG) against CgA, Syn, CXCL10, CXCL11 and HA, respectively. The blots were detected by the secondary antibody, horseradish peroxidase (HRP)-linked polyclonal goat anti-rabbit IgG for 1 hr at room temperature and Chemiluminescence reagents were used to visualize labeled proteins on X-ray film. GAPDH and α-tubulin were served as an internal loading control. All antibodies were diluted to 1:1000.

### RNA interference

The sequences of siRNA targeting human CgA cDNA and Syn cDNA were designed by Website of Life Technologies Company (http://rnaidesigner.life technologies.com/Rnaiexpress/setOption.do?designOption=sirna&pid=-6405575256610478702) and purchased from Shanghai GenePharma. The sequence of CgA siRNA was: 5′-GGGACAGUUCCAUGAAGCUTT-3′ and the non-inhibitory control siRNA was: 5′-GCUACAAGGAGAUCCGGAATT-3′; the sequence of Syn siRNA was sense: 5′-GCACCACCAAGGUCUUC UUTT-3′; and the non-inhibitory control siRNA was: 5′-GUACCGAGAGAAUAACAAATT-3′. The corresponding scrambled siRNA were transfected into colon cells in six-well plates using X-treme GENE siRNA Transfection Reagent (Roche, German) according to the manufacturer's instructions. Confirmation of silencing of target gene was measured by Western blotting 48h post-transfection.

### Cell invasion and boyden chamber assay

Transwell inserts were used to perform cell invasion assay. Cell migration was assessed using 24-well transwell cell culture chambers (8.0-lm pore polycarbonate membranes, Corning). After 1 day of serum starvation, the cells were harvested with trypsin and suspended in serum-free RPMI-1640. Subsequently, 10,000 cells in 200ul of serum-free RPMI-1640 were seeded into the upper chamber, and 500ul of RPMI-1640 containing 10% FBS was added to the lower chamber. The cells were allowed to migrate for 24 hr at 37°C in a 5% CO_2_ humidified atmosphere. Non-migrating cells were removed with a cotton swab. The filters were fixed in 4% paraformaldehyde and stained with Crystal Violet. Cell invasion assays were conducted using the same transwell cell culture chambers mentioned above except the filters were coated with 100 ug of matrigel. The invasion and migration assay procedures were the same. The cells that migrated and invaded through the filter were counted in six random 200x fields using a microscope.

### Q-PCR array

The ExProfile™ Gene qPCR Arrays are designed by GeneCopoeia (Inc, Rockville, Maryland, USA) for profiling the expressions of human chemokine & receptors. In each 96-well plate, there are up to 84 pairs of qPCR primers, showed in ESM S2, and 12 wells of controls which are used to monitor the efficiency of the entire experimental process – from reverse transcription to qPCR reaction.

### mRNA extraction and real time quantitative RT-PCR

Total RNA was extracted with trizol reagent (Invitrogen, Carlsbad, California, USA). cDNA was synthesized with the Prime Script RTase (Takara, Inc) according to the manufacturer's instructions. Real-time PCR for CgA, Syn, CXCL10, CXCL11, TYMP, ACKR4, CCRL2, GAPDH was performed on a LightCycler 480 system (Roche) using Premix Ex Taq (Takara, Inc) according to the manufacturer's instructions. The following primer pairs were used for each reaction: **Syn-F:** 5′-CTCGGCTTTGTGAAGGTGCT-3′ and **Syn–R:**5′-CTGAGGTCACTCTCGGTCTTG-3′; **CgA-F:**5′-TA AAGGGGATACCGAGGTGATG-3′and **CgA-R:**5′-TCG GAGTGTCTCAAAACATTCC-3′; **CXCL10-F:**5′-GTG GCATTCAAGGAGTACCTC-3′and **CXCL10-R:**5′-TGA TGGCCTTCGATTCTGGATT-3′; **CXCL11-F:**5′-GACG CTGTCTTTGCATAGGC-3′and **CXCL11-R:**5′-GGATT TAGGCATCGTTGTCCTTT-3′; **TYMP-F:**5′-GGTGTG GGTGACAAGGTCAG-3′and **TYMP-R:**5′-GCAGCAC TTGCATCTGCTC-3′; **ACKR4-F:**5′-GCCTTTTTGGGC TGTTAATG-3′and **ACKR4-R:**5′-TGATTGGCTGGGG ACTTTAG-3′; **CCRL2-F:**5′-AGCGATGAGGCAGAGC AATG-3′and **CCRL2-R:**5′-GGACACCGATCACAAAC ACAG-3′; **GAPDH-F**:5′-AGCCACATCGCTCAGACA C-3′and **GAPDH-R:** 5′-GAATTTGCCATGGGTGG A-3′.

### Statistics

Statistical analysis was performed using a SPSS software package (SPSS Standard version 13.0, SPSS Inc, USA). Differences between variables were assessed by the Chi-square test. All data are present as means±S.D. Survival analysis of patients with colorectal cancer was calculated by Kaplan-Meier analysis. A log rank test was used to compare different survival curves. Unpaired Student's t test and one way ANOVA were used as appropriate to assess the statistical significant of difference between two groups and three groups or more respectively. *P* Values <0.05 were considered significant.
